# Wellbeing Forecasting in Postpartum Anemia Patients

**DOI:** 10.3390/healthcare11121694

**Published:** 2023-06-09

**Authors:** David Susič, Lea Bombač Tavčar, Miha Lučovnik, Hana Hrobat, Lea Gornik, Anton Gradišek

**Affiliations:** 1 Department of Intelligent Systems, Jožef Stefan Institute, Jamova cesta 39, 1000 Ljubljana, Slovenia; 2 Jožef Stefan International Postgraduate School, Jamova cesta 39, 1000 Ljubljana, Slovenia; 3 Division of Gynaecology and Obstetrics, University Medical Centre Ljubljana, Šlajmerjeva ulica 3, 1000 Ljubljana, Slovenia; 4 Medical Faculty, University of Ljubljana, Vrazov Trg 2, 1000 Ljubljana, Slovenia

**Keywords:** wellbeing forecast, postpartum anemia, postpartum depression, fatigue, machine learning, Edinburgh Postnatal Depression Scale, Multidimensional Fatigue Inventory

## Abstract

Postpartum anemia is a very common maternal health problem and remains a persistent public health issue globally. It negatively affects maternal mood and could lead to depression, increased fatigue, and decreased cognitive abilities. It can and should be treated by restoring iron stores. However, in most health systems, there is typically a six-week gap between birth and the follow-up postpartum visit. Risks of postpartum maternal complications are usually assessed shortly after birth by clinicians intuitively, taking into account psychosocial and physical factors, such as the presence of anemia and the type of iron supplementation. In this paper, we investigate the possibility of using machine-learning algorithms to more reliably forecast three parameters related to patient wellbeing, namely depression (measured by Edinburgh Postnatal Depression Scale—EPDS), overall tiredness, and physical tiredness (both measured by Multidimensional Fatigue Inventory—MFI). Data from 261 patients were used to train the forecasting models for each of the three parameters, and they outperformed the baseline models that always predicted the mean values of the training data. The mean average error of the elastic net regression model for predicting the EPDS score (with values ranging from 0 to 19) was 2.3 and outperformed the baseline, which already hints at the clinical usefulness of using such a model. We further investigated what features are the most important for this prediction, where the EDPS score and both tiredness indexes at birth turned out to be by far the most prominent prediction features. Our study indicates that the machine-learning model approach has the potential for use in clinical practice to predict the onset of depression and severe fatigue in anemic patients postpartum and potentially improve the detection and management of postpartum depression and fatigue.

## 1. Introduction

Postpartum anemia is a very common maternal complication and remains a persistent public health issue globally, affecting up to 80% of women in developing countries [1]. The prevalence of anemia in women after childbirth remains unacceptably high in developed countries as well, with an estimated prevalence of up to 50% [2,3]. This is a condition where blood has a reduced capacity to carry oxygen to meet physiological needs due to low hemoglobin (Hb) concentration as a consequence of antepartum depletion of iron stores and/or excessive peripartum blood loss [2]. In comparison with pregnancy, maternal iron requirements usually decline during the puerperium beginning just after childbirth throughout the following six weeks [4]. This period, therefore, serves as a time to restore iron loss during pregnancy and childbirth since very little iron is excreted through breast milk [5].

Postpartum anemia is commonly defined by Hb values alone, with Hb level equal to or less than 100 g/L (10 g/dL) or <110 g/L at 1 week postpartum. Postpartum anemia could be diagnosed at any point during puerperium (the first six weeks after childbirth). Due to dilutional anemia, Hb values are lower than in non-pregnant women; e.g., first-trimester anemia is defined as Hb < 110 g/L vs. 120 g/L. The major causes of postpartum anemia are anemia during pregnancy combined with blood losses at delivery; therefore, postpartum anemia is defined by even lower Hb values (Hb < 100 g/L after childbirth) [6]. It is associated with several adverse health consequences, such as palpitations, dizziness, and breathlessness, and increases the risk of infections [2,7]. Additionally, from the psychological perspective, postpartum anemia may also adversely affect maternal mood, cognition, and behavior resulting in reduced physical and mental performance [3], including postpartum fatigue and depression [8,9,10,11,12] and reduced duration of breastfeeding [13]. All of these symptoms impair health-related quality of life, negatively affect the wellbeing of both the mother and child and significantly interfere with mother–child interactions [14,15,16], and can have serious negative implications for the health of the mother and the infant [17]. Postpartum depression occurs worldwide, with a prevalence of 5 to 15% [18]. Psychosocial risk factors for the development of depression and fatigue are well known [19], while we know less about the physiological factors. Research suggests that postpartum anemia is one of the latter. The incidence of postpartum depression and fatigue among anemic women is significantly higher compared to non-anemic women [9].

In most healthcare systems, women are hospitalized at the time of birth and discharged from the hospital within a few days. They are only seen again by healthcare professionals (midwives, obstetricians–gynecologists, or general practitioners) at the time of postpartum visit, usually six weeks postpartum [2]. Risks of postpartum depression are assessed by clinicians at the time of birth and in the days immediately following delivery by taking into account women’s psychosocial history and psychological factors such as postpartum Hb levels. Postpartum depression risk assessment is currently mostly conducted intuitively. As suicide remains one of the main causes of maternal mortality in developed countries, it stands to reason that the current clinical practice of identifying postpartum women at risk of developing depression is far from optimal [1].

In clinical practice, postpartum anemia—which is caused by loss of iron during pregnancy and/or loss of blood during childbirth [20]—is mostly treated by restoring iron stores. There are currently two main treatment approaches, iron supplementation administered orally or intravenously (IV) [2,6]. Most studies comparing these treatment approaches focused on laboratory values of various hematological parameters [21], resulting in a paucity of data on the impact of various iron treatments on actual clinical outcomes, such as the degree of maternal fatigue and depression.

As clinical practice typically sees a six-week gap between childbirth and the follow-up maternal visit, we investigated whether it is possible to train machine-learning models to forecast the patient status six weeks after birth based on the data at birth. Following a preliminary study on a smaller set of patients where we demonstrated that such models work reasonably well and outperform the baseline for prediction of ferritin levels, but less so for Hb [22], here, we focus on three parameters related to the patient wellbeing, namely depression, overall tiredness, and physical tiredness. Here, we should stress that our study focused on classical machine-learning approaches in order to provide models that are explainable. In comparison, deep learning approaches with neural networks often produce good classification models; however, they are essentially “black boxes”, providing no explanation of why a certain decision was reached. In medical applications of artificial intelligence (AI), explainable approaches are becoming increasingly important in view of trustworthy AI.

## 2. Materials and Methods

### 2.1. Dataset

The data used in this study were collected from a sample of 261 women, all white Caucasian, that were followed from pregnancy to six weeks postpartum. The data were collected by obstetricians–gynecologists at University Medical Centre Ljubljana. The features included in the data are given in Table 1. They include personal features (1–9,18), blood test features (11–17), survey-based features (19–21), and medication features [22]. Blood test features, BMI (body mass index), and survey-based features (11–21) were taken both right after birth as well as six weeks postpartum. The data did not include any missing values. Gestational age corresponds to the number of weeks since the last period. The type of childbirth is a categorical variable and can either be vaginal delivery or Cesarean section. Transfusion is a binary variable indicating whether a patient needs a blood transfusion after childbirth or not. Marital status is a categorical variable and can either be “lives alone”, “married”, or “non-marital partnership”. Education is an ordinal variable of 10 different values, with the lowest representing elementary school education and the highest representing a doctoral degree. Serum iron describes the amount of iron in the blood. Total iron-binding capacity (TIBC) is a good indicator of the amount of iron in the blood. If the iron level in the blood is low, the TIBC is higher as the free capacity for binding of the iron is higher. Transferrin saturation is the value of serum iron divided by the TIBC of the available transferrin. The higher the transferrin saturation, the bigger the iron stores in the body. C-reactive protein (CRP) is high if there is inflammation in the body. Inflammation can also be caused by an injury during childbirth or Cesarean section. Typically, CRP levels are increased after childbirth. Multidimensional Fatigue Inventory (MFI) covers several characteristics, one of which is physical tiredness, which was included as a separate feature. All patients were randomly administered iron supplementation either orally or intravenously. No placebo was used.

### 2.2. Methods

The aim of this study was to evaluate the performance of several classical machine-learning (ML) models in predicting the emotional distress of mothers with anemia in the postnatal period. The main outcome of the prediction was the EPDS score six weeks after childbirth. EPDS consists of ten questions, on the basis of which the mother’s wellbeing and the likelihood of postpartum depression are assessed [19]. Higher scores indicate a higher risk of postpartum depression in the mother. We also checked the performance of the ML models in predicting the MFI total score and the MFI physical tiredness score six weeks after childbirth. Scores were extracted from patient-reported fatigue symptoms measured by the Multidimensional Fatigue Inventory (MFI) questionnaire. It consists of 20 statements, for which the participant assesses the extent to which she has been subject to the particular aspect of fatigue in recent days. Higher scores indicate higher levels of fatigue. The MFI has been shown to be a good measure of health-related quality of life after birth [23].

For the predictor features, we used all features besides the ones collected six weeks after childbirth. The models were trained on a training set and tested on the test set. In the model training process, the models were trained to predict the outcome (EPDS score, MFI total score, or MFI physical tiredness score six weeks after childbirth) using the input features. To evaluate the models’ performance on previously unseen data, they were assessed on the test set. In order to compare the models, a 5-fold cross-validation approach was employed. This involved dividing the dataset into five equal parts, known as folds. Each fold was used as a testing set once, while the remaining four folds were combined to form the training set. This process was repeated five times, ensuring that each fold acted as a testing set exactly once. The results obtained from these five experiments were then averaged to obtain a comprehensive performance estimate. The cross-validation folds were stratified with respect to the administered medicine, ensuring that each fold maintained the same distribution of medication administration. Additionally, the models were compared to a baseline approach, which played the role of a reference point for the models’ performance. The baseline represents a simple and straightforward approach that serves as a point of comparison to assess the added value of more sophisticated models. In our case, the baseline approach always predicted the mean output of the train data. This type of baseline was used because the doctors currently have no clear way of determining the postpartum depression risk; thus, using the mean value of the train data is the most reasonable approach. 

We implemented nine ML regression models. These models can be grouped into three groups: linear models, support vector machines, and decision tree-based ensemble models. The linear models include linear regression (LR), kernel ridge (KR), elastic net regression (EN), and Bayesian ridge regression (BR) [24]. LR finds the best-fitting linear relationships between the predictor features and the output. KR is an extension of LR that includes a kernel function that also captures non-linear relationships. EN combines the strengths of both LR and KR to balance the trade-off between model complexity and sparsity. BR formulates linear regression using probability distributions rather than single-point estimates. Support vector regression (SVR) finds a non-linear hyper-plane in the feature space with the maximum margin to separate the data points. The decision tree-based ensemble models include gradient boosting regressor (GB), light gradient boosting machine (LGBM) [25], extreme gradient boosting regressor (XGB) [26], and catboost regressor (CB) [27]. GB builds an ensemble of decision trees by iteratively adding new trees that best fit the residual errors of the previous trees. LGBM is a more efficient implementation of GB that speeds up tree construction. XGB uses parallel and distributed computing to improve the performance of GB. CB employs techniques to better handle the categorical predictor features and to reduce overfitting.

We used mean absolute error (MAE), root mean squared error (RMSE), and R^2^ score as evaluation metrics, with MAE as the main metric. The formulas for the metrics are given in Equations (1)–(3), with *y_i_* representing the prediction, *x_i_* representing the true value, $\overset{-}{x}$ representing the mean true value, and *n* representing the total number of subjects. The R^2^ score of the model equals to 0 if the model always predicts the mean value of train data.

(1) $$\mathrm{MAE}=\frac{\sum_{i=1}^{n}\left|y_{i}-x_{i}\right|}{n}$$

(2) $$RMSE=\frac{\sqrt{\sum_{i=1}^{n}{\left(y_{i}-x_{i}\right)}^{2}}}{n}$$

(3) $$R^{2}=1-\frac{\sum_{i=1}^{n}{\left(y_{i}-x_{i}\right)}^{2}}{\sum_{i=1}^{n}{\left(y_{i}-\overset{-}{x}\right)}^{2}}$$

The results are reported as mean (standard deviation (SD), 95% confidence interval (CI)). The mean, SD, and 95% CI were calculated across results from all five folds.

## 3. Results

The results for the predictions of EPDS, total MFI score, and physical MFI score six weeks after childbirth are given in Table 2, Table 3 and Table 4 respectively. We see that in all three cases, all of the tested models outperformed the baseline according to MAE, and most of the models outperformed the baseline according to RMSE and R^2^ scores. The two best-performing models were EN and BR. To check the statistical significance of the results, we included the 95% CI of the results. We see that the best models in the prediction of EPDS six weeks after birth perform better than the baseline, even outside the bounds of the CI intervals. In the cases of predictions of MFI score six weeks after birth and physical MFI score six weeks after birth, we see that the models’ and baselines’ 95% CI partially overlap.

We also developed a binary classifier to predict whether the EPDS score lowers six weeks after childbirth (label 1) or not (label 0). The best-performing classifier was the support vector classifier, with an accuracy of 0.64 (0.02 SD, 0.61–0.68 95% CI). The baseline, which always predicted the mode of the train data, achieved an accuracy of 0.55 (0.03 SD, 0.51–0.59 95% CI). Again, the confidence intervals of both models do not overlap. 

As our dataset is relatively small, we did not perform any hyper-parameter tuning as we found that it can quickly result in overfitting even when using cross-validation. The values of the hyper-parameters used in our experiments were, therefore, the default values as per the Scikit-learn library [24]. 

The models’ training and performance evaluation was performed using Python 3.7 and libraries, Scikit-learn 0.24.2 [24], LightGBM 3.2.1 [25], XGBoost 1.4.2 [26], CatBoost 0.26 [27], and Numpy 1.18.5 [28].

### Investigating Predictor Features

While the forecasting models in all three cases outperform the baseline models, the models themselves do not give any particular information about why a certain decision was made. In medicine, it is an increasing trend to start using the models that are explainable, of so-called explainable artificial intelligence (XAI), where on the one hand, the practitioner understands why a model is behaving the way it is, and on the other hand, the explainable nature of the model can be used to generate new medical knowledge. With this approach, we look deeper into the case of the EPDS score.

**Figure 1:**
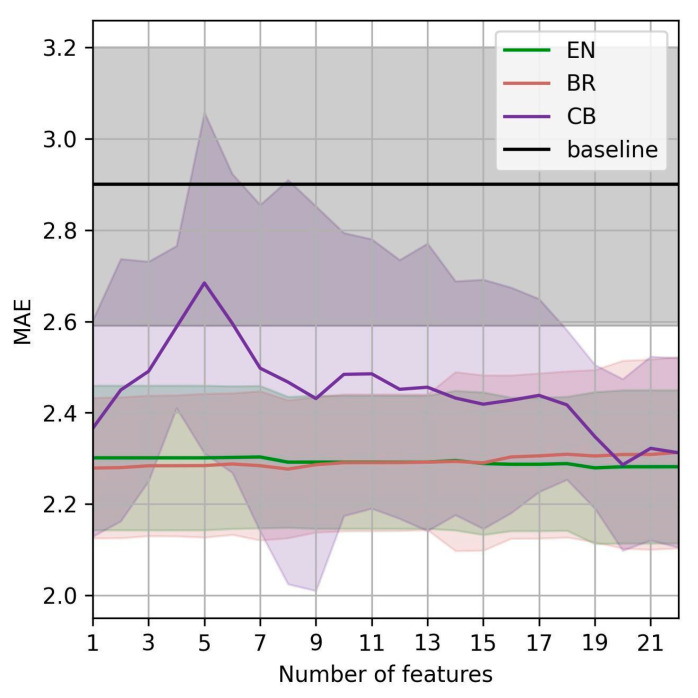
MAE of the best-performing models vs. number of predictor features for the prediction of EPDS six weeks after birth.

From the clinical perspective, gynecology experts prefer predicting the EPDS score six weeks after birth using as few predictor features as possible. We, therefore, investigated how the MAE of the best three models, EN, BR, and CB, changes with different predictor features. For this purpose, we first ranked the features from best to worst using mutual information [29] with the outcome. Starting with the most useful one, the most useful features turned out to be EPDS at birth, number of total childbirths, number of total pregnancies, marital status, phosphate level at birth, ferritin level at birth, education, MFI tiredness at birth, BMI at birth, and serum iron at birth. After the features were ranked, we measured the models’ performance by iteratively adding the features from the most to the least useful ones. The results are shown in Figure 1. We see that using 18 features, the top models outperform the baseline within the 95% CI. Additionally, we see that EN and BR outperform the baseline within the 95% CI using as little features as possible. The performance of those two models does not substantially improve with additional features; they perform very similarly using a single feature (EPDS as birth) using all 22 available features.

The type of iron supplementation (oral or IV) was initially expected to be an important feature in prediction models. However, as demonstrated, it does not appear among the features with substantial weight. Additionally, we separated the patients into two groups, IV and orally administered, and built models for each group separately. The improvements in view of MAE are marginal, and the models were trained on smaller datasets. Therefore, we do not investigate this approach further.

As the best-performing model, EN is a linear regression model, and we can check the coefficients of the fitted model. The coefficients explain the model-dependent feature importance for the prediction, each feature value is simply multiplied with the corresponding feature coefficient, and the sum of the multiplications across all features is the predicted output. The coefficients, given in Table 5, are averaged across all five folds.

Only eight (36%) of the features had a non-zero coefficient (when rounded to two decimal places), meaning all others are almost ignored by the model. Among those eight, only one feature (EPDS at birth) had a coefficient that was significant—all others are negligible (below 0.02 in absolute value). One possible conclusion could be that EPDS six weeks after birth could not be predicted using the features that are typically captured from pregnant women and mostly depends only on their current wellbeing at birth. After checking the Pearson coefficient between the predictor features and the EPDS six weeks after, we found that only three features had a score that was higher than 0.13 in absolute value: EPDS at birth (+0.56), MFI at birth (+0.32), and physical MFI at birth (+0.25).

## 4. Discussion and Conclusions

We look at the results from two perspectives, from the algorithm point and from the clinical usefulness point of view. In predicting all three wellbeing parameters, namely postpartum depression, overall tiredness, and physical tiredness, our models outperform the baseline in view of the mean average error and other metrics, albeit the improvement is not particularly large in the case of the EPDS score the best model has the MAE of 2.28, compared to the baseline of 2.9. Nevertheless, with the scores of patients in the study ranging from 0 to 19, such a range of errors has a practical use to clinicians, as it makes it clear to the doctor to see whether the patient is at risk of developing depression. It has to be noted that such risk assessment is currently conducted subjectively by the attending clinicians after birth. Therefore, a more objective and reliable method to predict postpartum depression risk would be very useful clinically, with the potential to prevent severe maternal morbidity and even mortality. The same can be said for the other two parameters: overall maternal fatigue and physical fatigue.

Looking at the parameters that show sizeable correlations with the wellbeing parameters six weeks after delivery, the main parameters are the wellbeing parameters recorded at childbirth. The fact that other parameters do not show prominent correlations and consequently do not appear (or appear with marginal weights) in the prediction models indicates that the patients in the dataset constitute a very heterogeneous group. A possible explanation of the results is that most of the parameters collected in the study simply do not significantly affect postpartum depression and fatigue. Other factors—perhaps sociological or genetic—could be more important and provide better prediction strength. This is a topic that should be considered more carefully in future research. 

While the best-performing model is of linear regression type, indicating that the EPDS score will typically decrease six weeks after delivery, this is not the case for all patients—in 85 (33%) patients, the score increased instead, and in 41 (16%) patients, the score did not change. To account for this, we developed a model that predicts in which direction the score will shift. This model again outperforms the baseline, although the accuracy of 0.64 is likely not yet sufficiently high for successful implementation into clinical practice with patient monitoring. Nevertheless, monitoring wellbeing parameters in patients with postpartum anemia is a rather new research topic. Thus, we believe that our results are relevant to the clinicians—especially in view of the fact that there is a six-week gap between the patient visits. To further improve the early detection of depression and maternal fatigue, additional checks for patients that appear prone to depression or maternal fatigue would be beneficial, allowing for better adjustment of treatment to improve the patient’s wellbeing.

## Figures and Tables

**Table 1:** Dataset features are either quantitative (Q), ordinal (O), or categorical (C). The mean and standard deviation (SD) of the quantitative features are given for both at birth (birth) and six weeks postpartum (after).

Index	Feature	Type	Mean (SD)
1	Age [years]	Q	31.4 (5.3)
2	Gestational age [weeks]	Q	38.5 (2.5)
3	Number of children born	Q	0.1 (0.2)
4	Number of total pregnancies	Q	1.8 (1.1)
5	Number of total childbirths	Q	0.6 (0.5)
6	Number of total abortions	Q	0.4 (0.7)
7	Type of childbirth	C	
8	Transfusion	C	
9	Marital status	C	
10	Education	O	
11	Haemoglobin [g/L]	Q	birth: 92.0 (5.9) after: 133.5 (8.0)
12	Serum iron [μmol/L]	Q	birth: 9.0 (6.4) after: 16.1 (6.1)
13	TIBC [μgmol/L]	Q	birth: 71.9 (10.1) after: 52.9 (8.4)
14	Transferrin saturation [%]	Q	birth: 12.6 (8.7) after: 30.8 (11.6)
15	Ferritin [μg/L]	Q	birth: 30.7 (27.8) after: 183.5 (145.9)
16	Phosphate [mg/dL]	Q	birth: 1.2 (0.2) after: 1.2 (0.2)
17	CRP [mg/L]	Q	birth: 64.6 (43.9) after: 1.7 (4.6)
18	BMI	Q	birth: 24.4 (4.4) after: 29.5 (4.5)
19	MFI	Q	birth: 50.2 (15.7) after: 37.9 (12.3)
20	MFI tiredness	Q	birth: 11.6 (4.1) after: 8.3 (3.3)
21	EPDS	Q	birth: 5.9 (3.9) after: 5.1 (3.5)
22	Medication	C	

**Table 2:** Results for the prediction of EPDS six weeks after birth. The values are reported as mean (SD, 95% CI).

Model	MAE	RMSE	R^2^
EN	**2.28 (0.12, 2.11–2.45)**	**2.97 (0.13, 2.78–3.15)**	**0.28 (0.08, 0.17–0.39)**
BR	2.31 (0.15, 2.10–2.52)	2.99 (0.15, 2.78–3.19)	0.27 (0.08, 0.16–0.39)
CB	2.33 (0.14, 2.14–2.52)	3.04 (0.20, 2.75–3.32)	0.25 (0.08, 0.14–0.36)
KR	2.37 (0.21, 2.08–2.67)	3.03 (0.18, 2.78–3.29)	0.25 (0.09, 0.12–0.38)
LGBM	2.38 (0.15, 2.17–2.59)	3.09 (0.17, 2.86–3.33)	0.22 (0.11, 0.07–0.37)
LR	2.40 (0.25, 2.06–2.75)	3.07 (0.23, 2.75–3.39)	0.23 (0.14, 0.03–0.43)
GB	2.46 (0.11, 2.31–2.62)	3.25 (0.19, 2.98–3.51)	0.14 (0.14, −0.06–0.34)
XGB	2.54 (0.28, 2.15–2.92)	3.29 (0.34, 2.81–3.76)	0.11 (0.19, −0.15–0.38)
SVR	2.68 (0.20, 2.40–2.97)	3.39 (0.26, 3.03–3.74)	0.07 (0.02, 0.05–0.10)
Baseline	2.90 (0.22, 2.59–3.20)	3.52 (0.27, 3.14–3.90)	0.00 (0.00, 0.00–0.00)

**Table 3:** Results for the prediction of total MFI score six weeks after birth. The values are reported as mean (SD, 95% CI).

Model	MAE	RMSE	R^2^
EN	**8.31 (0.64, 7.42–9.20)**	10.78 (0.80, 9.67–11.89)	0.20 (0.13, 0.02–0.38)
BR	8.34 (0.69, 7.38–9.30)	**10.74 (0.80, 9.62–11.85)**	**0.21 (0.11, 0.05–0.36)**
CB	8.64 (0.40, 8.09–9.19)	11.08 (0.46, 10.44–11.72)	0.15 (0.15, −0.06–0.36)
KR	8.72 (0.60, 7.89–9.56)	11.29 (0.71, 10.30–12.27)	0.12 (0.15, −0.09–0.32)
LR	8.79 (0.71, 7.81–9.77)	11.31 (0.76, 10.25–12.37)	0.11 (0.15, −0.09–0.32)
GB	8.90 (0.58, 8.10–9.70)	11.54 (0.58, 10.72–12.35)	0.08 (0.12, −0.08–0.25)
XGB	8.91 (0.48, 8.24–9.58)	11.63 (0.92, 10.35–12.91)	0.06 (0.20, −0.22–0.34)
LGBM	9.21 (0.29, 8.80–9.62)	11.81 (0.31, 11.38–12.24)	0.03 (0.19, −0.24–0.29)
SVR	9.30 (1.37, 7.40–11.19)	11.76 (1.38, 9.84–13.68)	0.06 (0.05, −0.01–0.14)
Baseline	10.04 (1.49, 7.97–12.12)	12.15 (1.32, 10.32–13.98)	0.00 (0.00, 0.00–0.00)

**Table 4:** Results for the prediction of physical MFI score six weeks after birth. The values are reported as mean (SD, 95% CI).

Model	MAE	RMSE	R^2^
EN	**2.35 (0.20, 2.07–2.62)**	**2.94 (0.28, 2.55–3.34)**	**0.16 (0.09, 0.02–0.29)**
BR	2.38 (0.21, 2.09–2.67)	2.97 (0.29, 2.56–3.37)	0.15 (0.08, 0.03–0.26)
KR	2.41 (0.19, 2.15–2.68)	3.06 (0.29, 2.66–3.46)	0.08 (0.15, −0.13–0.29)
LR	2.43 (0.20, 2.16–2.70)	3.08 (0.30, 2.67–3.49)	0.07 (0.15, −0.14–0.29)
CB	2.43 (0.17, 2.20–2.66)	3.07 (0.25, 2.72–3.41)	0.08 (0.09, −0.05–0.21)
SVR	2.47 (0.24, 2.13–2.81)	3.01 (0.33, 2.56–3.47)	0.12 (0.06, 0.03–0.21)
LGBM	2.47 (0.22, 2.17–2.78)	3.19 (0.22, 2.89–3.49)	0.00 (0.11, −0.15–0.16)
GB	2.48 (0.18, 2.23–2.73)	3.17 (0.27, 2.80–3.55)	0.02 (0.11, −0.13–0.17)
XGB	2.64 (0.13, 2.46–2.82)	3.38 (0.21, 3.08–3.67)	−0.12 (0.15, −0.33–0.09)
Baseline	2.70 (0.37, 2.18–3.21)	3.22 (0.35, 2.73–3.71)	0.00 (0.00, 0.00–0.00)

**Table 5:** Coefficients of the EN model for predicting the EPDS score. The values are reported as mean (SD, 95% CI).

Feature	EN Coefficient
EPDS at birth	0.42 (0.03, 0.38–0.46)
Education	0.01 (0.01, −0.01–0.03)
MFI tiredness at birth	0.01 (0.02, −0.02–0.04)
ITM at birth	−0.01 (0.01, −0.03–0.00)
Transferin saturation	−0.01 (0.00, −0.02–0.01)
MFI at birth	0.02 (0.01, 0.01–0.03)
TIBC at birth	0.02 (0.00, 0.02–0.03)
Gestational age	−0.01 (0.02, −0.03–0.01)

## Data Availability

Data will be made available upon request.

## References

[1] Milman N. (2011). Postpartum anemia I: Definition, prevalence, causes, and consequences. Ann. Hematol..

[2] Milman N. (2012). Postpartum anemia II: Prevention and treatment. Annal. Hematol..

[3] Bergmann R.L., Richter R., Bergmann K.E., Dudenhausen J.W. (2010). Prevalence and risk factors for early postpartum anemia. Eur. J. Obstet. Gynecol. Reprod. Biol..

[4] Simoes E., Kunz S., Bosing-Schwenkglenks M., Schmahl F.W. (2004). Maternal postpartum anaemia–tendencies and variability, considering different hospital categories–research on the basis of perinatology in Baden-Württemberg. Z. Geburtshilfe Neonatol..

[5] Shashiraj F.M.M., Singh O., Rusia U. (2006). Mother’s iron status, breastmilk iron and lactoferrin—Are they related?. Eur. J. Clin. Nutr..

[6] Pavord S., Myers B., Robinson S., Allard S., Strong J., Oppenheimer C., British Committee for Standards in Haematology (2012). UK guidelines on the management of iron deficiency in pregnancy. Br. J. Haematol..

[7] Oppenheimer S.J. (2001). Iron and its relation to immunity and infectious disease. J. Nutr..

[8] Corwin E.J., Murray-Kolb L.E., Beard J.L. (2003). Low hemoglobin level is a risk factor for postpartum depression. J. Nutr..

[9] Lee K.A., Zaffke M.E. (1999). Longitudinal changes in fatigue and energy during pregnancy and the postpartum period. J. Obstet. Gynecol. Neonatal. Nurs..

[10] Beard J.L., Hendricks M.K., Perez E.M., Murray-Kolb L.E., Berg A., Vernon-Feagans L., Tomlinson M. (2005). Maternal iron deficiency anaemia affects postpartum emotions and cognitions. J. Nutr..

[11] Sheikh M., Hantoushzadeh S., Shariat M., Farahani Z., Ebrahiminasab O. (2017). The efficacy of early iron supplementation on postpartum depression, a randomized double-blind placebo-controlled trial. Eur. J. Nutr..

[12] Holm C., Thomsen L.L., Nørgaard A., Langhoff-Roos J. (2017). Single-dose intravenous iron infusion or oral iron for treatment of fatigue after postpartum haemorrhage: A randomized controlled trial. Vox Sang..

[13] Rioux F.M., Savoie N., Allard J. (2006). Is there a link between postpartum anemia and discontinuation of breastfeeding?. Can. J. Diet Pract. Res..

[14] Bodnar L.M., Cogswell M.E., McDonald T. (2005). Have we forgotten the significance of postpartum iron deficiency?. Am. J. Obstet Gynecol..

[15] Ando K., Morita S., Higashi T., Fukuhara S., Watanabe S., Park J., Hotta T. (2006). Health-related quality of life among Japanese women with iron-deficiency anemia. Qual. Life Res..

[16] Moya E., Phiri N., Choko A.T., Mwangi M.N., Phiri K.S. (2022). Effect of postpartum anaemia on maternal health-related quality of life: A systematic review and meta-analysis. BMC Public Health.

[17] Troy N.W. (2003). Is the significance of postpartum fatigue being overlooked in the lives of women?. MCN Am. J. Matern. Child Nurs..

[18] O’Hara M.W., McCabe J.E. (2013). Postpartum depression: Current status and future directions. Annu. Rev. Clin. Psychol..

[19] Beck C.T. (2002). Theoretical perspectives of postpartum depression and their treatment implications. MCN Am. J. Matern. Child Nurs..

[20] Breymann C. (2015). Iron deficiency and anaemia in pregnancy: Modern aspects of diagnosis and therapy. Eur. J. Obstet Gynecol..

[21] Sultan P., Bampoe S., Shah R., Guo N., Estes J., Stave C., Butwick A.J. (2019). Oral vs intravenous iron therapy for postpartum anemia: A systematic review and meta-analysis. BJOG.

[22] Susič D., Bombač Tavčar L., Hrobat H., Gornik L., Lučovnik M., Gradišek A. (2022). Detection of postpartum anemia using machine learning. Proceedings of the 25th International Multiconference.

[23] Jansen A.J., Essink-Bot M.L., Duvekot J.J., van Rhenen D.J. (2007). Psychometric evaluation of health-related quality of life measures in women after different types of delivery. J. Psychosom. Res..

[24] Pedregosa F., Varoquaux G., Gramfort A., Michel V., Thirion B., Grisel O., Duchesnay E. (2011). Scikit-learn: Machine Learning in Python. JMLR.

[25] Ke G., Meng Q., Finley T., Wang T., Chen W., Ma W., Liu T.Y. Lightgbm: A highly efficient gradient boosting decision tree. Proceedings of the 31st International Conference on Neural Information Processing Systems (NIPS’17).

[26] Chen T., Guestrin C. XGBoost: A Scalable Tree Boosting System. Proceedings of the 22nd ACM SIGKDD International Conference on Knowledge Discovery and Data Mining (KDD’16).

[27] Prokhorenkova L., Gusev G., Vorobev A., Dorogush A.V., Gulin A. CatBoost: Unbiased Boosting with Categorical Features. Proceedings of the 32nd International Conference on Neural Information Processing Systems (NIPS’18).

[28] Harris C.R., Millman J.K., van der Walt S.J., Gommers R., Virtanen P., Caurnapeau D. (2020). Array programming with numpy. Nature.

[29] Kraskov A., Stögbauer H., Grassberger P. (2004). Estimating mutual information. Phys. Rev. E.

